# Continuous Intraperitoneal Insulin Infusion for People With Type 1 Diabetes: A Literature Review and International Position Statement

**DOI:** 10.1111/dom.71054

**Published:** 2026-07-09

**Authors:** Nick Oliver, Bernhard Gehr, Rayhan Lal, Peter R. van Dijk, Eric Renard

**Affiliations:** ^1^ Department of Metabolism, Digestion and Reproduction Imperial College London London UK; ^2^ Center for Diabetology and Metabolism Bad Heilbrunn Germany; ^3^ Department of Medicine, Division of Endocrinology Stanford University Stanford California USA; ^4^ Department of Pediatrics, Division of Endocrinology Stanford University Stanford California USA; ^5^ Diabetes Center Isala Zwolle the Netherlands; ^6^ Montpellier University Hospital, Department of Endocrinology and Diabetes and Institute of Functional Genomics, CNRS, INSERM University of Montpellier Montpellier France

**Keywords:** glycaemic control, hypoglycaemia, insulin pump therapy, insulin therapy

## Abstract

Achieving glucose targets without hypoglycaemia is the treatment goal in type 1 diabetes. Structured education, intensified insulin injection regimens, continuous glucose monitoring, automated insulin delivery, and ongoing support from a multidisciplinary team all support people with type 1 diabetes to achieve this goal. Despite these advances, significant barriers to achieving optimal management remain. Continuous intraperitoneal insulin infusion has comparable or better glucose outcomes to continuous subcutaneous insulin infusion and may reduce the frequency of hypoglycaemia, including severe episodes. Intraperitoneal insulin may be considered as a treatment modality for children and adults with type 1 diabetes using optimised intensive insulin therapy for whom subcutaneous insulin has failed due to lipoatrophy, ‐dystrophy or ‐hypertrophy, local allergy, subcutaneous insulin resistance or co‐existing skin conditions. Failure of subcutaneous insulin may result in recurrent or unexplained severe hypoglycaemia or hyperglycaemia. Intraperitoneal insulin may also be considered as a treatment modality for people with type 1 diabetes with severe needle‐phobia, and for those being considered for islet cell or pancreatic transplantation, or where transplantation is not available. This paper summarises current intraperitoneal insulin delivery technology, its potential risks and benefits, and an expert position statement. It is intended for use by diabetes specialist healthcare professionals, and as a reference for other healthcare professionals, commissioners, payors, people with diabetes, their carers, and advocates.

## Executive Summary

- For adults and children with type 1 diabetes on optimised intensive therapy, intraperitoneal insulin should be considered where subcutaneous insulin is unsuitable due to one of the following clinical conditions:
  ○ Congenital or acquired lipodystrophy, lipoatrophy, or lipohypertrophy.
  ○ Cutaneous allergy to insulin preparations or to materials used to deliver insulin.
  ○ Subcutaneous insulin resistance.
  ○ Co‐existing skin conditions preventing subcutaneous insulin use.
- Intraperitoneal insulin therapy may be considered for people with type 1 diabetes treated by subcutaneous insulin therapy with recurrent or unexplained severe hypoglycaemia or hyperglycaemia despite maximal optimisation, who may otherwise be considered for islet or pancreas transplantation.
- Intraperitoneal insulin therapy should be provided in a multidisciplinary specialist type 1 diabetes centre.

## 1 Introduction

Currently, type 1 diabetes almost universally requires lifelong treatment with insulin. Intensive insulin treatment, aiming for normal or near normal circulating glucose concentrations reduces the risk of micro‐ and macrovascular complications over time but is associated with an increased risk of hypoglycaemia, which may be severe and has significant impact on the quality of life of a person with type 1 diabetes [1, 2, 3].

Clinically, insulin is delivered through the intravenous, subcutaneous, intramuscular, inhaled or intraperitoneal routes and is, most commonly, self‐administered subcutaneously. Differing insulin regimens may be used according to personal preference to match lifestyle, diet, activity and employment, and include long‐acting insulin to provide a basal circulating insulin concentration, and rapid‐acting insulin to match ingested carbohydrate in a multiple dose injection (MDI) regimen as a baseline therapy. Compared to MDI regimens of subcutaneous insulin, continuous subcutaneous insulin infusion (CSII) can support people with type 1 diabetes to achieve a lower HbA1c with an associated reduction in the frequency and severity of hypoglycaemia, and increased food freedom. This makes CSII an effective treatment choice for people who are unable to achieve target glycaemia without disabling hypoglycaemia [4]. The combination of continuous glucose monitoring with CSII (and possibly even with glucagon) in a closed loop configuration further lowers haemoglobin A1c (HbA1c), increases time spent in the target range and reduces the time spent in hypoglycaemia [5]. However, while an optimised HbA1c without hypoglycaemia is the target of type 1 diabetes glucose management it is not always achievable with an intensified subcutaneous insulin regimen, and severe hypoglycaemia remains prevalent, even with HCL systems [6, 7, 8, 9]. This review summarises the current evidence and provides expert position statement for continuous intraperitoneal insulin infusion therapy in type 1 diabetes.

## 2 Continuous Intraperitoneal Insulin Infusion

### 2.1 Physiology

In people with intact pancreatic islets, insulin is secreted directly into the portal circulation and has its first action on the liver. This is critically important for glucose homeostasis, maintaining glycogen storage, glycogenolysis and gluconeogenesis. The first phase of insulin secretion seen with an oral carbohydrate load has the important effect of switching off hepatic gluconeogenesis, active in the fasted state, to prevent elevated macronutrient concentrations (including glucose) post‐prandially. In type 1 diabetes this first phase insulin secretion into the portal circulation is lost and post‐prandial hyperglycaemia can be challenging. Subcutaneous insulin delivery results in higher peripheral circulating concentrations of insulin but does not result in the higher portal concentrations seen with pancreatic secretion [10]. Strategies to overcome this include giving prandial insulin 15 min or more before a meal to allow time for the administered insulin to be absorbed and have a hepatic impact and, for people using CSII, the ‘superbolus’, where the prandial insulin and next 2 h basal insulin are all given in advance of a meal then the pump is suspended for 2 h, maximising initial concentrations of portal insulin to inhibit gluconeogenesis [11]. Furthermore, the sustained peripheral hyperinsulinemia of long‐term subcutaneous insulin is associated with insulin resistance, potentially exacerbating cardiometabolic risk [12].

Delivering insulin through the intraperitoneal (IP) route mitigates the subcutaneous absorption delay and ensures insulin is delivered to the portal circulation through the capillaries of the visceral peritoneum [13, 14, 15]. Insulin administered through the IP route is detectable within the portal system within 1 min of delivery and peak action is reached after 15 min [13, 16]. Even with state‐of‐the‐art rapid acting insulin, the onset of action of a subcutaneous bolus is around 20 min after administration and peak action can be between 1 and 3 h after injection [17] depending on the type and dose of rapid acting insulin. After IP insulin administration post‐prandial blood glucose peaks are lower with reproducible and more predictable insulin profiles compared to subcutaneous (SC) insulin [16, 18, 19, 20].

The proportion of IP insulin which is absorbed by the hepatic portal system is unknown, though indirect evidence suggests that it is considerable in type 1 diabetes [21]. Data from animal studies with IP and SC insulin demonstrate that peripheral insulin levels are 68% higher with the SC route, although the total insulin requirement is the same for both IP and SC routes. In addition, hepatic glucose output is 45% higher with SC insulin while, for a similar level of glycaemic control, the IP route achieves peripheral insulin concentrations similar to those observed in non‐diabetic animals [10]. The potential clinical benefit in reducing hepatic glucose output may be to reduce circulating glucose concentrations, especially post‐prandially. Additionally, reducing peripheral insulin concentration may reduce the risk of hypoglycaemia and cardiometabolic sequelae [12].

In addition to direct portal insulin delivery and reduced circulating peripheral insulin concentrations, IP insulin is also associated with a restoration of glucagon and growth hormone secretion and enhances hepatic glucose production in response to hypoglycaemia and activity [22, 23, 24, 25, 26]. The mechanism for this is unclear but may be, in part, attributable to lower intra‐islet insulin concentrations acting on the alpha cell, increased hepatic sensitivity to glucagon, or hepatic glucose utilisation during hypoglycemia [22, 26]. Restoration of glucagon and growth hormone secretion has a significant potential clinical benefit in reducing exposure to hypoglycaemia in type 1 diabetes.

### 2.2 Clinical Studies of the Impact of IP Insulin on Glucose

**FIGURE 1:**
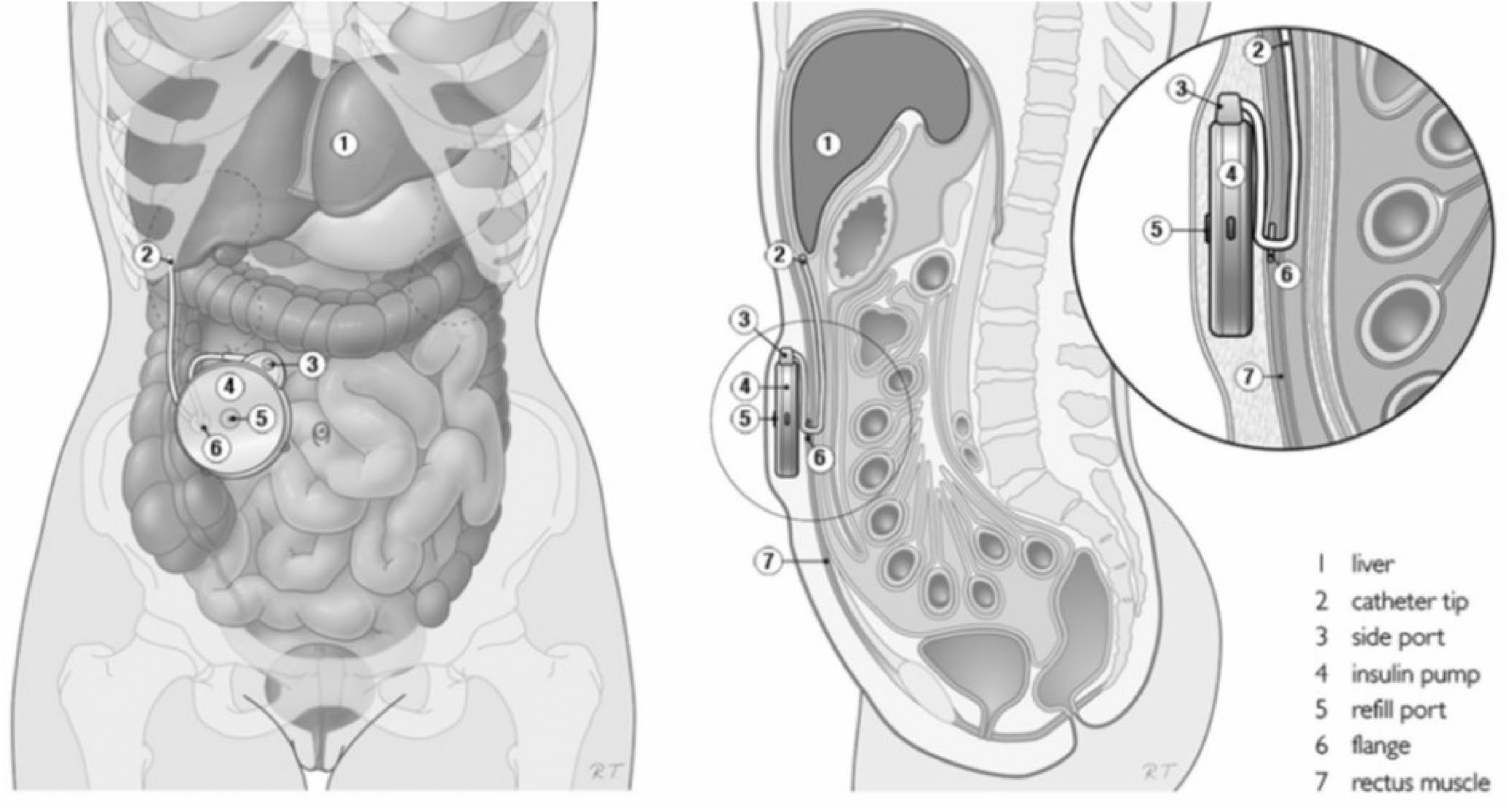
Implanted intraperitoneal insulin pump. Reproduced from van Dijk et al. [27].

Four randomised controlled studies have assessed the effect of intraperitoneal insulin delivery compared with continuous subcutaneous insulin or a MDI regimen in people with type 1 diabetes. Three of these studies compared an implanted IP pump system (Medtronic or Infusaid, Figure 1) against subcutaneous insulin [28, 29, 30] and one study compared an implanted IP catheter (DiaPort, Roche Diabetes) and continuous intraperitoneal insulin infusion against CSII [31]. To date, to our knowledge, no randomised controlled studies have been conducted compared to hybrid closed loop subcutaneous insulin delivery.

In the mentioned studies HbA1c fell with intraperitoneal insulin by between 0.6% and 1.2% (7–13 mmol/mol), and reductions in incident hypoglycaemia (capillary glucose less than 3.5 mmol/L) and in the incidence of severe hypoglycaemia were seen compared to subcutaneous insulin (see Table 1). In the DiaPort study the incidence of severe hypoglycaemia was significantly lower with the IP catheter system compared with CSII [31]. Participants with frequent hypoglycaemia on CSII were randomised to CSII or continuous IP insulin and, while the frequency of any hypoglycaemia was the same between the two groups (IP 118.2 (SD 82.6) events/patient year, CSII 115.8 (SD 75.7) *p* = 0.910), the incidence of severe hypoglycaemia with IP insulin was half that observed in the CSII group (IP 34.8 events/100 patient years, CSII 86.1, *p* = 0.013). A meta‐analysis of continuous intraperitoneal insulin infusion (CIPII) reports a 0.61% (6.7 mmol/mol) mean reduction in HbA1c compared with CSII, with reduced exposure to severe and non‐severe hypoglycaemia [32]. Heterogeneity of study design and duration, and differences in data presentation limit the strength of the analysis but the data included are comprehensive and, where possible, outcomes are well standardised.

**TABLE 1:** IP insulin type 1 diabetes study summary.

Trial	Study design	*n*	Duration	Device	Endpoint	Hypoglycaemia	Outcome
Selam 1992	Randomised controlled trial with CIPII and CSII arms	21	3 months	Infusaid 1000	Glycated Hb between group difference	Severe and biochemical (< 3.4 mmol/L) reported	Equivalent reduction in GHb and hypoglycaemia seen in both groups
Broussolle 1994	Multicentre prospective observational	224	353 patient years	Infusaid 1000 (*n* = 48), Minimed MIP 2001 (*n* = 205), Promedos ID3 (*n* = 7)	Glycated Hb	Severe events fell from 15.2 to 2.5 per 100 patient years (*p* < 0.001)	HbA1c fell from 7.4% (57 mmol/mol) to 6.8% (50 mmol/mol)
Haardt 1994	Randomised controlled trial CIPII and CSII arms	10	6 months	Infusaid 1000, Minimed MIP 2001	Cost benefit	Mild and severe events recorded	Greater HbA1c, mild hypoglycaemia, and SD fall in IP group. 2.6 fold higher direct costs with IP
Hanaire‐Broutin 1995	Multicentre prospective observational	224	353 patient years	Infusaid 1000 (*n* = 48), Minimed MIP 2001 (*n* = 205), Promedos ID3 (*n* = 7)	Safety and feasibility	No change in frequency of glucose < 3.5 mmol/L. Severe events fell from 15.2 to 2.5 per 100 patient years (*p* < 0.001)	CIPII abandoned in *n* = 11; 47 catheter obstruction events; 9 pump failures
Jeandidier 1996	Multicentre prospective observational. Persistent CIPII users compared with subcutaneous	240 (*n* = 120 persistent CIPII), *n* = 120 subcutaneous following CIPII cessation	214 patient years (CIPII group); 51 patient years (SC group)	Not described	Severe hypoglycaemia	0.11 events per patient year (CIPII); 0.69 events per patient year (SC) *p* < 0.001	Lower frequency of severe hypoglycaemia in group that persisted with CIPII
Logtenberg 2009	Randomised crossover trial CIPII and SC (CSII or MDI) arms	24	6 months	Medtronic MIP 2007c	Primary: incidence of hypoglycaemia Secondary: HbA1c, time in range	No between group differences in hypoglycaemia	Improved glucose control and time in target with CIPII compared with SC insulin
Liebl 2009	Randomised crossover trial CIPII and CSII arms	60	6 months	Accu‐Chek DiaPort	Primary: Frequency of glucose < 3 mmol/L Secondary: Severe hypoglycaemia, HbA1c, DQoL	Frequency of glucose < 3 mmol/L was not different between groups. Severe hypoglycaemia significantly lower with CIPII (*p* = 0.013)	HbA1c not different between groups. DQoL better with CIPII
Van Dijk 2014	Prospective follow up following randomised crossover trial CIPII and CSII arms	19	6 years	Medtronic MIP 2007c	Long‐term efficacy, safety and quality of life	Incidence of glucose < 3.5 mmol/L reduced with CIPII	Treatment satisfaction greater with CIPII
Van Dijk 2014	Matched control observational study	95 (*n* = 21 CIPII, *n* = 74 SC)	7 years	Medtronic MIP 2007c	Long‐term impact of CIPII	Incidence of hypoglycaemia decreased over time with CIPII compared with SC	No other differences seen between groups

In addition to these randomised trials, several observational studies have been reported with both technologies [33, 34, 35, 36, 37, 38, 39]. These publications show similar improvements in HbA1c with continuous subcutaneous and IP insulin delivery and a trend towards a lower incidence of hypoglycaemia with IP insulin. However, all of the studies were in small populations and were not powered to demonstrate changes in hypoglycaemia rates.

The pharmacokinetics of IP insulin and hypoglycaemia protection make it the ideal route for a fully closed loop insulin delivery system compared to subcutaneous insulin, potentially removing the requirement for meal and exercise announcements. In a pilot study of 10 adults with type 1 diabetes, IP insulin with a fully closed loop system without meal announcement was compared to SC insulin in two clinic visits lasting 24 h, including 3 unannounced meals of 70, 40 and 70 g of carbohydrate. The IP route led to significantly greater time in range (3.9–10 mmol/L) with no difference in exposure to hypoglycaemia. Additionally, more insulin could be delivered at mealtimes with the IP route than with the SC route, addressing post‐meal glucose excursions without inducing hypoglycaemia [40].

### 2.3 Clinical Studies of the Impact of IP Insulin on Non‐Glucose Outcomes

Differences in responses in other metabolic pathways have been observed with IP compared with SC insulin delivery. These are hypothesised to be primarily related to the higher portal insulin concentrations and the lower peripheral concentrations. IP insulin in several studies has been observed to increase biologically active Insulin‐like Growth Factor 1 (IGF‐1) by both increasing IGF‐1 itself and by reducing Insulin‐like Growth Factor Binding Protein 1 (IGFBP‐1) concentrations [41, 42, 43, 44, 45]. Growth hormone axis dysregulation has been described in type 1 diabetes and may have a role in hypoglycaemia risk and macrovascular complications [46, 47] and data from a prospective, observational case–control study suggest that adults using IP insulin have a higher calcification propensity time than those on SC insulin. A shorter time is associated with a greater propensity to calcify, and has been independently associated with atherosclerotic events in people with chronic kidney disease [48].

Compared to SC insulin, IP insulin also reduces hepatic sex hormone binding globulin (SHBG) production by around 20% (with a greater effect seen in women), leading to higher concentrations of free sex hormones [49], and has an effect on lipid metabolism by inhibiting hepatic very low density lipoprotein (VLDL) production and activating hepatic lipase [50]. IP insulin is associated with an increase in triglyceride concentration compared with SC insulin with a reduction or no change in high density lipoprotein (HDL)‐cholesterol, but small numbers of subjects and short duration of follow up limit the interpretation of these data [50, 51, 52]. The high HDL state seen with subcutaneous insulin administration presents a paradox as type 1 diabetes is associated with an excess cardiovascular risk despite apparently favourable concentrations of HDL. The normalisation of lipid profiles seen with intraperitoneal insulin may, therefore, have beneficial effects on cardiovascular risk, with further longitudinal studies needed to elucidate this.

### 2.4 Quality of Life

In all studies of people starting IP insulin, improvements in self‐reported quality of life, diabetes‐specific quality of life, treatment satisfaction or diabetes worry have been described, suggesting a positive impact of IP insulin on people with type 1 diabetes using CSII and unable to achieve target glucose [31, 53, 54]. It should be noted that these improvements were noted in both the randomised and observational studies, suggesting an effect over and above that of patient choice, but studies of IP insulin are not blinded. In both the observational and randomised studies, the people who use CIPII often represent a very selected group who already have a poorer quality of life, relative to the people on subcutaneous insulin with whom they are compared. Taking that into account, it is all the more striking that CIPII leads to an improvement in quality of life.

### 2.5 Technology

Implanted intraperitoneal pumps (Figure 1) are implanted in the deep fascia overlying the rectus abdominus muscle with a catheter extending into the peritoneal space. They contain a reservoir which can be filled transcutaneously, usually with U400 human regular insulin, and the pump can be programmed with a wireless control unit. Device reservoir capacity is 15 mL (6000 units), increasing to 20 mL (8000 units) in new generation devices. Surgical implantations under general anaesthesia are performed exclusively by certified centres that are currently located mainly in Europe (France and The Netherlands).

The DiaPort system (Roche Diabetes, Germany) consists of an indwelling intraperitoneal catheter connected to a percutaneous port implanted in the anterior abdominal wall. A standard insulin infusion pump (Roche Accu‐Chek Combo) connects to the port and is operated as for CSII. U100 human regular insulin is used with the DiaPort device (Figure 1b). The DiaPort system has been unavailable since the end of 2025 due to economic considerations by the manufacturer and a strategic reorientation including withdrawal from the insulin pump market. Existing DiaPort users will continue to be supplied with consumables for as long as possible.

### 2.6 Proposed Indications

The evidence base supporting IP insulin is limited by comprising a small number of studies in restricted cohorts [14, 15, 16, 17, 18, 19, 20, 21, 22, 23]. Proposed indications for IP insulin therapy are, first, based on data demonstrating a benefit for people with type 1 diabetes where subcutaneous insulin has failed due to problems with absorption (see summary box). This may be due to an identifiable condition such as lipoatrophy, lipohypertrophy, a congenital or acquired lipodystrophy, some rare skin diseases [55] or severe cutaneous allergy to insulin or the components of delivery. Some people may present with ‘subcutaneous insulin resistance’, which has been defined as a subcutaneous insulin: intravenous insulin dose ratio of 3:1 or greater [56]. Other indications may include recurrent or unexplained severe hypoglycaemia or hyperglycaemia despite hybrid closed loop (HCL) systems and in people for whom islet or pancreatic transplantation is indicated but not possible or is not the person's choice, and, finally, where there is persistent severe needle‐phobia despite psychological intervention, to reduce self‐management needle burden and limit the use of needles to a supervised setting. Severe hypoglycaemia is defined as an episode of hypoglycaemia requiring 3rd party assistance for treatment [57], and recurrent is 2 or more episodes per year.

IP insulin has been used off‐label safely and effectively, usually in older children over the age of 12 [58]. There is no known minimum age or weight for use in children and further data are required in paediatric and adolescent age groups.

### 2.7 Contraindications

Continuous intraperitoneal insulin infusion is not compatible with peritoneal dialysis therapy. An increased incidence of circulating anti‐insulin antibodies has been reported with IP insulin, with variable metabolic consequences from none to the ‘low morning syndrome’, characterised by recurrent nocturnal hypoglycaemia contrasting with post‐meal hyperglycaemia. It is not clear if antibody positivity is a relative contraindication to IP insulin therapy [59, 60]. When considering intraperitoneal insulin, it is important to consider assessment of anti‐insulin antibody titres before proceeding.

People with multiple previous abdominal surgeries may not be candidates for IP insulin because, to be effective, it requires a functioning peritoneal surface area for absorption. A detailed surgical history and multidisciplinary assessment with an expert surgical team are therefore critical.

### 2.8 Alternative Therapies

Alternative treatment modalities for people with type 1 diabetes where CIPII may be a treatment option include SC hybrid closed‐loop insulin delivery systems, continuous intravenous insulin therapy, islet cell transplantation, and pancreas transplantation. Continuous intravenous insulin therapy requires long‐term intravenous access with the associated risk of sepsis [61] and thrombosis and requires significant education, support, and healthcare professional input. Furthermore, there are no approved intravenous insulin pump systems for out‐of‐hospital personal use leading to the necessity of ‘off‐label‐use’. It can be, however, an effective glucose management modality [61, 62].

Islet cell transplantation is associated with an improvement in glycaemic control and a reduction in severe hypoglycaemia, with preserved graft secretion of insulin over several years. However, it is limited for several reasons: it does not usually lead to insulin independence, it may require multiple pancreata for each recipient with significant sensitisation, and it is costly—and therefore not available in many countries. Also, it is generally limited to patients with good renal function (eGFR > 60 mL/min), an insulin requirement of less than around 0.7 units/kg/day and to those who are able to accept the risks of immunosuppression (drug side effects, malignancy and severe infection) [63]. Pancreas transplantation is a major surgical undertaking with a significant operative risk and a large economic cost. In common with islet cell transplantation, there is a requirement for long‐term immunosuppression and long‐term graft survival is lower than for renal transplantation.

Given the reversible nature of IP insulin and its comparatively low risks and costs when compared with transplantation and intravenous insulin, it may be a treatment modality to consider as an alternative to these therapies, particularly in people with higher insulin requirements, or it may be a safe and effective bridging therapy while awaiting transplantation.

### 2.9 Insulin

Insulin administered by the intraperitoneal route differs from that used subcutaneously or intravenously in that only human soluble insulin is used in combination with a stabilising agent to reduce the risk of insulin aggregation and line occlusion. Rapid acting insulin analogues are not suitable for IP administration and have been associated with catheter occlusion. At present, the only proprietary insulin preparation recommended for use in intraperitoneal delivery systems is U400 Insuman Implantable (Sanofi, Frankfurt, Germany) for use in implanted pumps and U100 Insuman Infusat (Sanofi, Frankfurt, Germany) for use in the DiaPort system. Insuman is unique in containing polyethylene‐polypropylene glycol (Poloxamer 171) as a stabilising agent to reduce the risk of catheter occlusion [64, 65]. More concentrated and stable insulins are under development.

### 2.10 Setting

Continuous intraperitoneal insulin therapy should be delivered by a multidisciplinary specialist type 1 diabetes team including a consultant diabetologist, surgical support from a consultant‐led team experienced in managing IP insulin systems and their surgical complications, IP insulin trained diabetes specialist educators, dietitian, and a diabetes specialist psychologist. At the point of determining eligibility for CIPII and during treatment, thorough psychological assessment and support are very important. People on this clinical pathway often have a long history of complex diabetes with associated, or resulting, distress, anxiety or mood disorders. Sometimes these are not apparent at the outset but emerge later and may be a barrier to effective therapy. Intraperitoneal insulin therapy should be delivered in a centre with access to appropriate alternative therapies and with high levels of experience of managing people with complex diabetes, including severe hypoglycaemia, subcutaneous insulin absorption problems, and with links to specialist dermatology.

### 2.11 Clinical Pathway for Intraperitoneal Insulin

**FIGURE 2:**
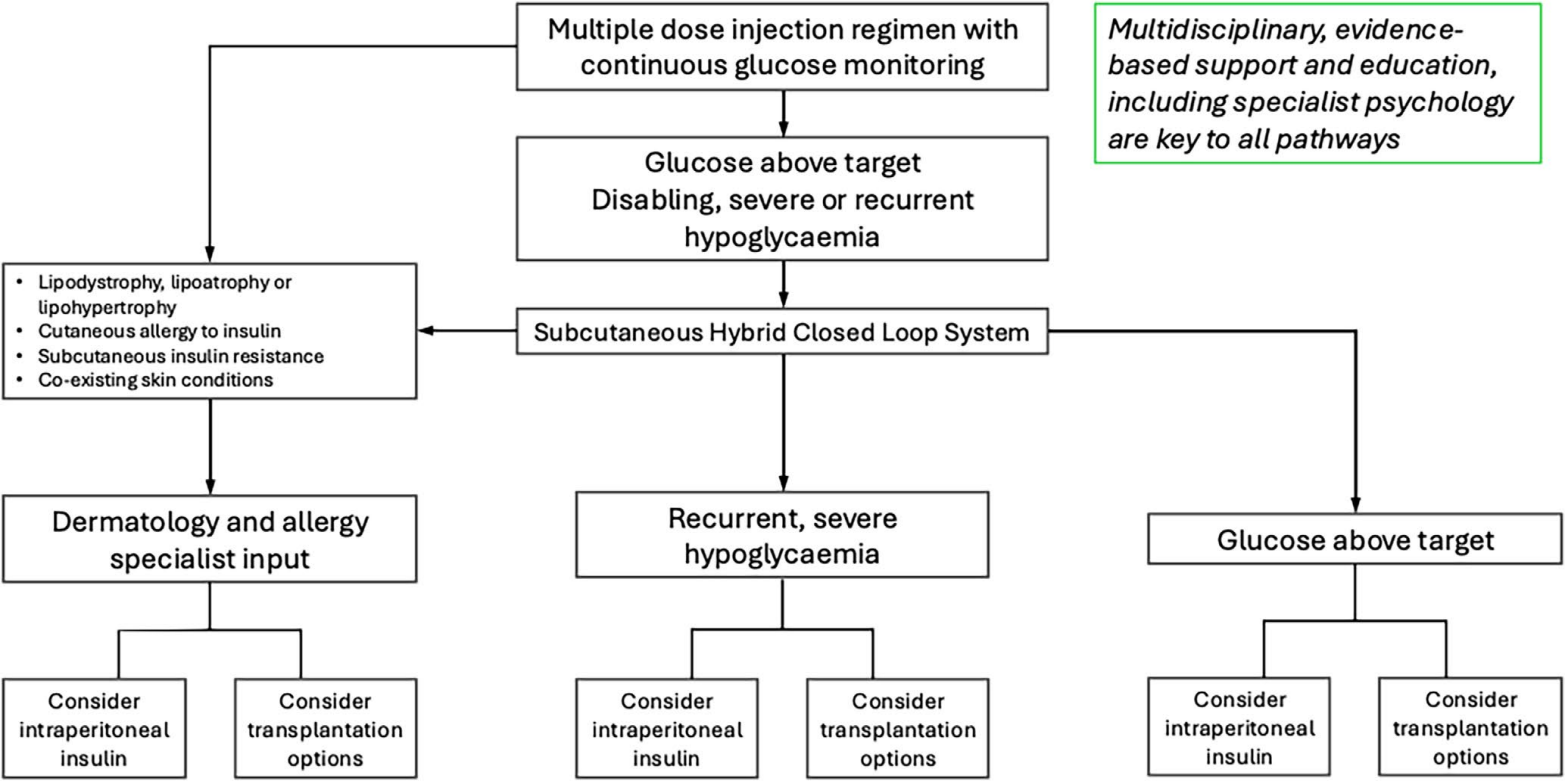
Clinical pathway for intraperitoneal insulin.

A pathway for people with type 1 diabetes is shown in Figure 2. Throughout this pathway, education, support, and psychological intervention are critical components of multidisciplinary input. Major barriers to optimising glucose self‐management may not be addressed by intraperitoneal insulin, making specialist psychology support and access to appropriate intervention therapies critical.

In the proposed clinical pathway people with type 1 diabetes on MDI therapy, supported by continuous glucose monitoring, with challenging hypo‐ or hyperglycaemia progress to hybrid closed loop. Once established, if recurrent or severe hypoglycaemia remains present, islet cell or pancreas transplantation may be discussed if unacceptable hypoglycaemia awareness, frequency or severity remain [66]. People with type 1 diabetes and unexplained recurrent severe hypoglycaemia not responding to optimised care may also be considered for intraperitoneal insulin therapy.

If, while using subcutaneous insulin, an insulin absorption defect is clinically suspected based on insulin requirement or skin features, manoeuvres to address this should be considered. Insulin delivery and absorption should be carefully reviewed with consideration of common issues affecting insulin absorption such as lipohypertrophy or lipoatrophy. Alternative cannulae, insulins and infusion sites may be considered. A structured approach to people with diabetes requiring high insulin doses (> 2 units/kg/day) has been published and is important to follow [67], and dynamic protocols exist to investigate subcutaneous insulin absorption (Supporting Information). These are based on assessing the difference in glucose lowering action between a variable rate intravenous insulin infusion and subcutaneous dosing. Insulin and insulin receptor antibody status may also be investigated, and inherited and acquired lipodystrophies may be considered. Confirmation of a subcutaneous insulin absorption issue, or where there is a dermatological diagnosis or unsuitability for transplantation should prompt consideration of intraperitoneal insulin. People with type 1 diabetes and unexplained recurrent severe glucose deviations requiring inpatient care may also be considered for intraperitoneal insulin therapy as a modality to reduce debilitating glucose variability resulting in an impact on quality of life and high care costs [68].

### 2.12 Risks

IP insulin pump systems require a surgical procedure for insertion with associated risks of anaesthesia, bleeding, infection and discomfort. During use the most common complications of continuous intraperitoneal insulin infusion therapy are catheter occlusion (8.1 per 100 patient years), pump dysfunction (4.2 per 100 patient years), pain (3.9 per 100 patient years) and local infection with the DiaPort system (2.5 per 100 patient years) [69]. The root cause for IP catheter obstruction has been shown to be a foreign body reaction [70]. However, 80% of people using IP insulin had no reported pump complications over a 15‐month period. Peritonitis is rare, occurring 0.4 per 100 patient years and no mortality from peritonitis has been reported with IP insulin. Aseptic peritonitis has been reported in rare cases [71]. The presently reported median time between implantation and further surgical intervention is 4.5 (95% CI: 4.1–4.8) years [69]. The median time from implantation to the occurrence of the first complication requiring operative intervention was 3.6 years (95% CI: 2.2–5.0 years).

### 2.13 Cost

The cost for an implantable pump is around €30 000 with an expected battery life duration of 7 years. Additional costs include outpatient visits for pump reservoir filling every 6 weeks and the cost of U400 insulin. Costs for comparable therapies are around €80 000 for a single islet transplant in Europe [72] and $290 000 for a pancreas transplant in the United States (2011 billed cost estimate) [73]. However, the 5‐year survival of pancreas grafts in simultaneous pancreas kidney transplantation in the UK is 86% (95% CI 83%–89%), and the 5‐year graft survival following the first islet transplant is 61% (95% CI 48%–72%) [74].

## 3 Conclusion

After optimal standard multidisciplinary care, intraperitoneal insulin delivery is a treatment option for people with type 1 diabetes when SC insulin is either an unsuitable therapy or it has failed to achieve safe or effective glucose management. It may also be a suitable treatment option for people with type 1 diabetes and severe hypoglycaemia despite optimised SC insulin with ongoing multidisciplinary education and support, and for whom transplantation options are either unavailable, unsuitable, or undesirable. Intraperitoneal insulin has the potential to improve HbA1c, to reduce the burden of hypoglycaemia, and has additional effects on hepatic, lipid and endocrine pathways which may have benefits, especially for cardiovascular risk. The evidence supporting this position statement remains limited and further studies are required, in particular focusing on the potential benefits of IP insulin for hypoglycaemia and cardiovascular risk, compared with optimal standard care [75].

## Funding

N.O. reports infrastructure support for this research was provided by the NIHR Imperial Biomedical Research Centre (BRC).

## Conflicts of Interest

N.O. has received consultation and/or lecture fees from Eli Lilly and Company, Dexcom, Medtronic, Roche Diabetes, AstraZeneca, Sanofi, Tandem, and has received research support from Dexcom, Medtronic, and Roche Diabetes.

B.G. declares consultant/speaker fees from Abbott, Ascensia, Dexcom, Diabeloop, Insulet, Lilly, Novartis, NovoNordisk, Medtronic, Roche, Sanofi, Theras, and Vitalaire.

R.L. has received consulting fees from Abbott Diabetes Care, Adaptyx Biosciences, Biolinq, Capillary Biomedical, Deep Valley Labs, Gluroo, Portal Diabetes, Sanofi, and Tidepool. He has served on advisory boards for Provention Bio, Lilly, and Rezolute. He receives research support from his institutions from Insulet, Medtronic, Sinocare, and Tandem.

P.R.D. declares consultant/speaker fees from A. Menarini Diagnostics, Abbott, Medtronic, and research support from A. Menarini Diagnostics, Abbott, Dexcom Inc., and Medtronic.

E.R. declares consultant/speaker fees from A. Menarini Diagnostics, Abbott, Air Liquide SI, Astra‐Zeneca, Becton‐Dickinson, Boehringer‐Ingelheim, Cellnovo, Dexcom Inc., Eli‐Lilly, Hillo, Insulet Inc., Johnson & Johnson (Animas, LifeScan), Medtronic, Medirio, Novo‐Nordisk, Roche, and Sanofi‐Aventis and research support by Abbott, Dexcom Inc., Insulet Inc., Roche, and Tandem Diabetes Care.

## Data Availability

Data sharing not applicable to this article as no datasets were generated or analysed during the current study.
